# Advances in Real-Time 3D Reconstruction for Medical Endoscopy

**DOI:** 10.3390/jimaging10050120

**Published:** 2024-05-14

**Authors:** Alexander Richter, Till Steinmann, Jean-Claude Rosenthal, Stefan J. Rupitsch

**Affiliations:** 1 Fraunhofer Institute for High-Speed Dynamics, Ernst–Mach–Institut (EMI), Ernst-Zermelo-Straße 4, 79104 Freiburg, Germany; 2 Electrical Instrumentation and Embedded Systems, Albert–Ludwigs–Universität Freiburg, Goerges-Köhler-Allee 106, 79110 Freiburg, Germany; till.steinmann@imtek.uni-freiburg.de (T.S.); stefan.rupitsch@imtek.uni-freiburg.de (S.J.R.); 3 Fraunhofer Institute for Telecommunications, Heinrich–Hertz–Institut (HHI), Einsteinufer 37, 10587 Berlin, Germany

**Keywords:** computer vision, minimally invasive surgery, medical endoscopy, real-time 3D reconstruction

## Abstract

This contribution is intended to provide researchers with a comprehensive overview of the current state-of-the-art concerning real-time 3D reconstruction methods suitable for medical endoscopy. Over the past decade, there have been various technological advancements in computational power and an increased research effort in many computer vision fields such as autonomous driving, robotics, and unmanned aerial vehicles. Some of these advancements can also be adapted to the field of medical endoscopy while coping with challenges such as featureless surfaces, varying lighting conditions, and deformable structures. To provide a comprehensive overview, a logical division of monocular, binocular, trinocular, and multiocular methods is performed and also active and passive methods are distinguished. Within these categories, we consider both flexible and non-flexible endoscopes to cover the state-of-the-art as fully as possible. The relevant error metrics to compare the publications presented here are discussed, and the choice of when to choose a GPU rather than an FPGA for camera-based 3D reconstruction is debated. We elaborate on the good practice of using datasets and provide a direct comparison of the presented work. It is important to note that in addition to medical publications, publications evaluated on the KITTI and Middlebury datasets are also considered to include related methods that may be suited for medical 3D reconstruction.

## 1. Introduction

**Figure 1:**
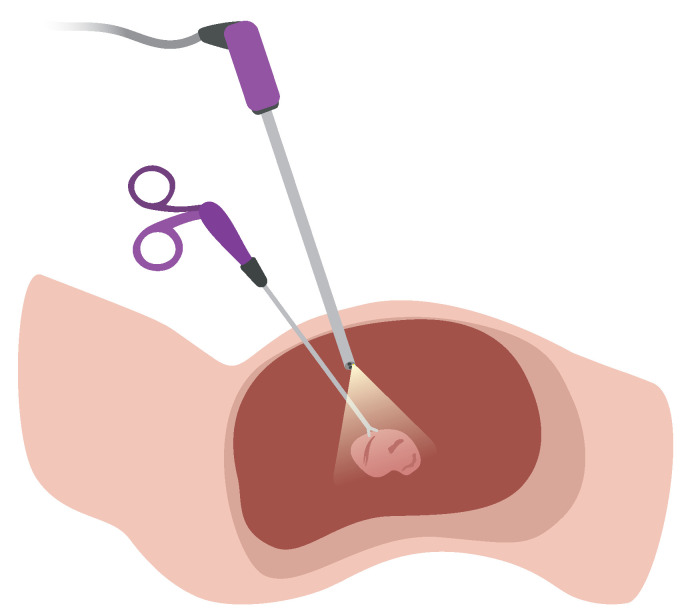
Exemplary depiction of a laparoscopic MIS, also referred to as keyhole surgery, using an endoscope.

In recent decades, Minimally Invasive Surgery (MIS) has demonstrated superior patient recovery and surgical outcomes with smaller incisions, less pain, lower risk of infection, reduced blood loss, shorter hospital stays, and less scarring than traditional surgery. In the context of laparoscopy, which is depicted in Figure 1, substantial progress was made concerning image quality, in vivo imaging techniques such as narrow-band, intra-operative tools in robotics, 3D position tracking, 3D reconstruction of surgical scenes, and many more [1,2]. Despite this progress, a well-experienced surgeon is still needed to carry out minimally-invasive interventions due to a multitude of reasons: a small field of view, a restricted range of motion, a shadowless illumination of the surgical area, and a lack of depth perception, to name a few. But even for trained professionals, it is hard to accurately survey the correct dimensions of anatomical landmarks [3].

During MIS only limited visual cues for depth perception are visible. This increases mental rotation and transformation challenges to surgeons and can contribute to misjudgment, increased cognitive workload, and fatigue [4]. This is underlined by an evaluation of 252 cases of laparoscopic cholecystectomy procedures, where 97% of surgical accidents occurred as a result of visual misperceptions [5]. When compared to conventional MIS imaging, higher-resolution 3D imaging systems have been shown to significantly improve the depth perception of surgeons, which is critical for the precision and safety of surgical procedures [3,6].

By further taking into account the depth information of a scene and creating a 3D reconstruction in real-time, it becomes possible, e.g., to determine the distance between two points more precisely. There are various applications for real-time 3D reconstructions, missing region detection in colonoscopy, registration in sinus surgery, and laparoscopic surgery. Together with Augmented Reality (AR), 3D reconstruction allows for highlighting and viewing objects or organs from different points of view. During a bronchoscopy, colonoscopy, or sinus surgery, a 3D reconstruction could give the physician orientation by utilizing AR to show 3D objects as landmarks, which makes it easier to place stents or remove tissue from the correct location. 3D reconstructions could also provide depth information to cancer- or polyp-detecting algorithms to provide more information and help better detect cancer tissue.

The real-time capability of 3D reconstruction algorithms is essential for surgeries where the physician can be assisted during an operation. In this case, the algorithm has to provide depth information promptly. If the already present video feed during an operation can be used to analyze the scene in real-time and provide depth information without hindering the physician, the optional depth information can only be beneficial. Additional assistance, e.g., by offering navigational aid or removing surgical instruments from the surgeon’s view is also conceivable, although instrument-tissue interaction makes gathering depth information in these areas challenging [7]. Moreover, navigation and instrument segmentation or removal are not in the scope of this contribution.

In principle, it is possible to perform a 3D reconstruction with greater detail in a post-operation video analysis, then however, the generated depth information is not available during the operation. Especially when considering that modern videoendoscopes such as the TIPCAM®3D videoendoscope from KARL STORZ SE & Co. KG, Tuttlingen, Germany, are equipped with 4K resolution at 60 Hz. As of today, due to computational limitations, the real-time constraint does introduce challenges concerning the amount of data that can be processed and is largely dependent on the used hardware. Hence, we focus on publications that claim real-time functionality at the time of publication and those that are likely to reach real-time functionality, with state-of-the-art hardware.

This contribution compares real-time 3D reconstruction methods and the used datasets for evaluation, and further highlights the importance of common evaluation techniques. Common evaluation techniques allow for a comparison between contributions and thereby make advances in the field of real-time 3D reconstruction for medical endoscopy visible. Many of the reviewed contributions use their own evaluation metrics and set of images, which makes it impossible to rank them in the current state-of-the-art. In some datasets, the evaluation parameters for 3D reconstructions are well-defined; for self-sampled images, these parameters are not often published.

Fortunately, new computer vision and AI-based algorithms in the fields of autonomous driving, robotics, and unmanned aerial vehicles have attracted attention for their success in tracking and reconstructing scenes in real time. However, most developed methods rely on a true-to-scale representation of their environment and assume a non-deformable “static world”. In contrast, this assumption does not hold for surgical applications, where organic tissue is inevitably deformed, especially when using flexible endoscopes, where independent and simultaneous movement of the endoscope and tissue is possible. Nevertheless, these advancements could pave the way for groundbreaking achievements in the domain of surgical intra-operative assistance.

**Figure 2:**
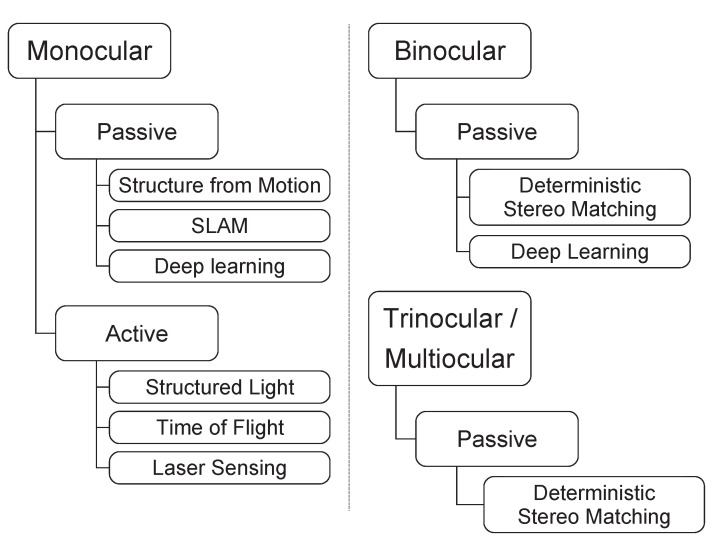
Overview of the state-of-the-art real-time camera-based acquisition systems for 3D reconstruction that are discussed in this contribution.

As researchers steadily compete to find the best solutions, various approaches for surgical intra-operative assistance have been developed. Some of the methods presented in our work require additional hardware to function. Others solely rely on the visual input of the image sensor and, therefore, do not require further modifications to the existing MIS hardware. To provide a comprehensive overview of the current state-of-the-art for real-time 3D reconstruction methods, a thematic division of the discussed contributions is realized in Figure 2. Here, first, the number of lenses is categorized. Then, passive and active methods are distinguished, and while active methods actively emit a measurand to perform a 3D reconstruction, passive methods rely only on an image sequence provided by the image sensor. Please note that the categorization is performed as provided by the original authors.

While other categorizations such as Endoscope Flexibility, Application Environment, or Operational Complexity are possible; here, we focus mainly on the technologies used for 3D reconstruction. Nevertheless, it is important to be aware of the characteristics of each technology when examining the results presented in this review. For example, flexible endoscopes can have only one lens and a considerably shorter working distance than rigid endoscopes.

Under closer inspection, it becomes apparent that it is challenging to directly compare the sheer endless number of methods in detail. The problems here vary from missing information, e.g., execution times or used hardware, to non-published self-constructed evaluation setups that prevent a comparison against established methods. Therefore, in the following sections, let us contrast the most relevant real-time contributions concerning the state-of-the-art. In this contribution, we concur with the statement of Liu et al. [8] and define real-time for MIS applications as 10 Frames per Second (FPS) or above.

## 2. Evaluation Tools

This chapter outlines the tools used to compare different 3D reconstruction methods. Methods are compared under the consideration that parameters such as the field of view, and distance to the object can influence the 3D reconstruction. In medical applications, the observed scenes can be challenging as they can contain structureless surfaces, challenging lighting conditions, and liquids, like blood.

First, we will discuss the good practice of using datasets and the associated benefits, such as the ability to evaluate and compare algorithms against each other objectively. For this, both medical and non-medical datasets are addressed.

### 2.1. Disparity Map vs. Point Cloud

The two primary forms of representing 3D data of stereoscopic scenes are the disparity map and the point cloud [9]. The disparity map is a pixel-based inverse representation of the scene’s depth range from a given perspective [10]. The disparity range in an image can be calculated, for example by Confidently Stable Matching [11], or statistical analysis of the spatial correlation between stereo images [12]. The point cloud is a set of 3D data points that represent the captured scene in 3D space. A conversion between both is possible using the intrinsic and extrinsic camera parameters obtained from a calibration process [13]. To evaluate the accuracy of an algorithm, disparity maps, and point clouds can both be used to compare a result against ground truth data.

Camera-based 3D reconstruction systems commonly exploit the disparity between two images to create a disparity map. However, not all vision systems rely on disparities for depth perception. The time-of-flight technique can obtain depth information directly by measuring the flight time of emitted directional light while measuring occurring phase shifts [14]. Knowing the speed of light and the direction of the emitted light, results in a 3D coordinate, thus directly creating a point cloud.

### 2.2. Metrics for Comparison

**Figure 3:**
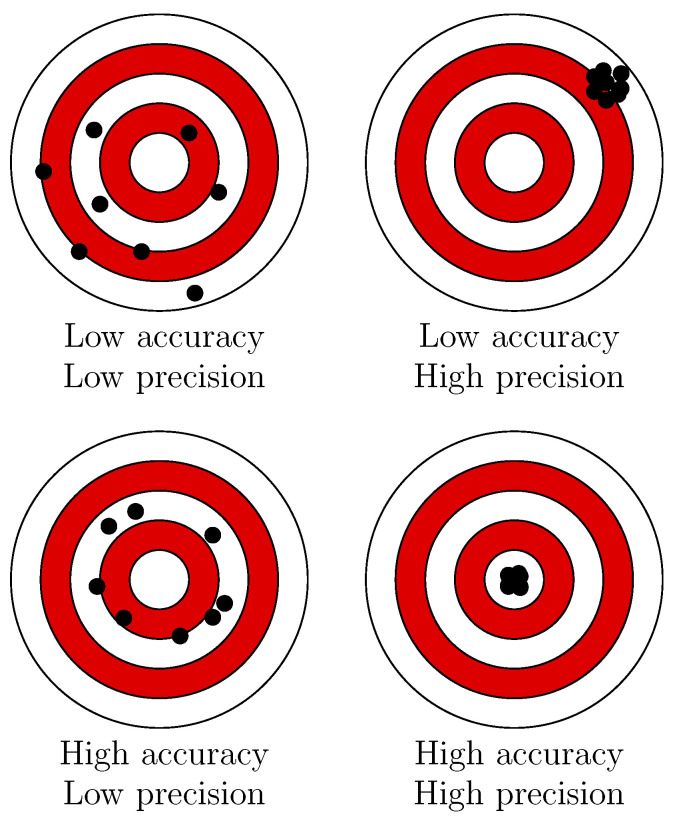
Illustration of accuracy vs. precision, where the bullseye represents the true value that is expected, while black dots represent measurements, hence throwing results.

It is essential to differentiate between accuracy and precision, as they are sometimes incorrectly used interchangeably. Accuracy denotes how well the mean value of multiple measurements fits the expected value of the measured quantity. Precision describes how far the measurements are scattered from their mean value on average. For example, when shooting darts at a target, the bullseye is the expected value of the observed throws. The closer the average of all hits is to the bullseye, the higher the accuracy. The closer the hits are to each other, regardless of the location, the higher the precision. This matter is illustrated in Figure 3.

Concerning 3D reconstruction, high precision is often assumed if the algorithm repeatedly produces the same result when evaluating a single image. A better consideration in terms of accuracy, however, is the analysis concerning all pixels of an image. Then, the precision represents the ability to reproducibly deliver results with the same accuracy, and the accuracy represents how true to detail the 3D reconstruction is concerning the ground truth data. Therefore, when a new method is introduced, both the precision and the accuracy should be stated. In the following paragraphs, we aim to introduce the metrics used in the reviewed literature.

One of the easiest methods to evaluate the quality of a 3D reconstruction against ground truth data is to compare the disparity maps, which are described in Section 2.1. Assuming a disparity map produced from an arbitrary stereo reconstruction algorithm and a ground truth disparity map, ideally representing the true disparity map without any errors, then, the mean absolute error (MAE) between the 3D reconstruction and the ground truth data can be calculated according to

(1) $$\mathrm{MAE}=\frac{\sum_{p=1}^{n}\left|{\hat{y}}_{p}-y_{p}\right|}{n},$$

where ${\hat{y}}_{p}$ is the pixel at position *p* in the resulting disparity map of an arbitrary algorithm, $y_{p}$ is the pixel at position *p* in the ground truth disparity map, and *n* is the number of pixels in each disparity map. The MAE is a measure of the average magnitude of errors in a test set, without considering their direction. It describes the equally weighted average over all absolute differences between the expected result and the actual result. This unit of measure is used, for example, when comparing different algorithms against the Stereo Correspondence and Reconstruction of Endoscopic Data (SCARED) dataset by Allan et al. [15].

One of the most significant error metrics is the root mean squared error (RMSE) [16], which is used in the Stereo-Endoscopic Reconstruction Validation dataset based on conebeam CT (SERV-CT) dataset [17] and is defined by

(2) $$\mathrm{RMSE}=\sqrt{\frac{\sum_{p=1}^{n}{\left({\hat{y}}_{p}-y_{p}\right)}^{2}}{n}}.$$

The RMSE describes the square root of the average of squared differences between the expected result and the actual result. Since the errors are squared, the RMSE gives a higher weight to large errors [18]. Therefore, the RMSE penalizes larger errors more harshly. This becomes clear when comparing five imaginary measurements with an evenly distributed error of two, which results in an MAE and RMSE of 2. However, if the same measurement is performed with four correct values and only one outlier of value ten, we will obtain an MAE of 2 and an RMSE of roughly 4.5. In both cases, the total error is ten, yet the RMSE is higher for the second case due to the larger deviation from the expected value.

**Figure 4:**
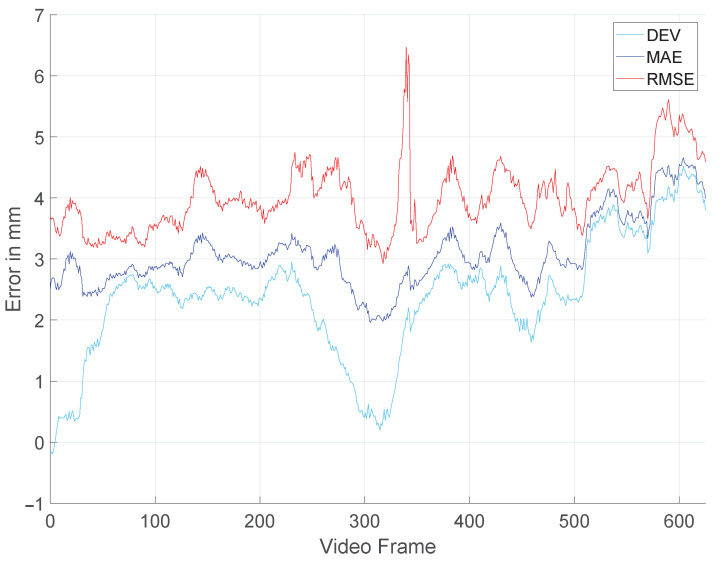
Visual representation of commonly used error metrics when comparing a method’s performance against ground truth data of a dataset. Where DEV is the deviation to the ground truth, and MAE and RMSE as defined in Section 2.2.

To illustrate the differences between the error metrics, Figure 4 demonstrates an exemplary evaluation for each metric. It is important to note that the same evaluation data are used for each error metric.

Figure 4 clearly shows that the error metrics are not interchangeable and the curves show a distinct behavior. Depending on the application, one might choose the MAE metric over the RMSE or vice versa. The MAE is less sensitive to outliers compared to the RMSE [18]. Therefore, to better understand how true to detail a 3D reconstruction is, the MAE might be better suited. However, to determine the accuracy of a model under the consideration of outliers, the RMSE is the better choice.

Assuming that the error distribution is somewhat Gaussian, it is good practice to provide the standard deviation (SD) in addition to the MAE or RMSE. The standard deviation (SD) is defined by

(3) $$\mathrm{SD}=\sqrt{\frac{\sum_{p=1}^{n}{\left({\hat{y}}_{p}-\mu \right)}^{2}}{n-1}},$$

where μ is the mean of ${\hat{y}}_{p}$ over all pixels *p*. The SD is a measure of the amount of variation of a set of values and denotes the precision.

Some datasets introduce error metrics to determine the accuracy of a disparity map or point cloud. The *Middlebury* dataset (MD) employs a metric, which defines the error, as the percentage of pixels whose absolute disparity error is larger than one pixel. An alternative to MD’s D1-all error is defined by the *Karlsruhe Institute of Technology and Toyota Technological Institute* (KITTI) dataset. It calculates the percentage of pixels with an estimation error larger than 3 pixels and larger than 5% of the true disparity [19]. The Bad3 error is similar to the D1-all error metric of the KITTI dataset, as it determines the percentage of pixels with an estimation error larger than 3 pixels. It is used in the SERV-CT dataset to calculate the accuracy of the alignment to the true point cloud.

The runtime of an algorithm is another factor that is especially important for real-time applications. In the field of computer vision, typically either the execution time measured in seconds or the FPS are provided. Depending on the application, the number of times an algorithm must run per second can vary. Many factors influence the FPS of a 3D reconstruction algorithm. Larger depth ranges and larger image sizes, for example, can increase the runtime of an algorithm [20]. In addition, the underlying hardware also has a large impact on the performance, which is discussed further in Section 2.5.

The correct classification of these metrics plays a fundamental role in objective comparability. As many factors influence the performance of an algorithm in terms of quality and speed, the best approach to objectively evaluate and compare new methods is to use common public datasets with consistent metrics. This fact is elaborated in Section 2.3.

### 2.3. Datasets

**Figure 5:**
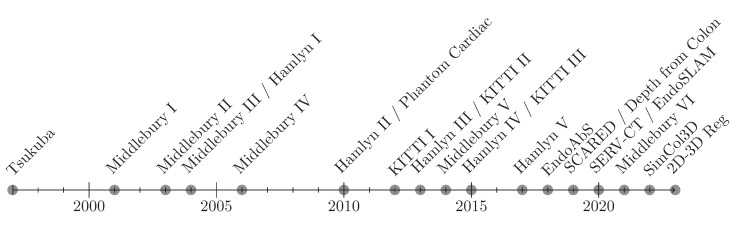
Historic timeline of datasets for 3D reconstruction.

Historically, the comparison of methods targeting 3D real-world problems or “3D in the wild” has always been a big challenge in the computer vision community. To overcome this, strong and continuous efforts have been made starting in the late 1990s, introducing the most famous Tsukuba stereo test image dataset with known ground truth values. Nowadays, datasets from various publishers can be easily found on the web. An overview of the datasets used by contributions mentioned in this publication is presented in Figure 5 and Table 1. The significant advantage of using datasets is the ability to objectively and independently compare developed methods against each other by guaranteeing that all contributions use the same data and error metrics. Ideally, the raw data and ground truth data are both provided. In the field of 3D reconstruction that could be a video of a dynamic scene or a still stereo image paired together with a ground truth point cloud.

Some of the less recent datasets use smaller image sizes and are therefore difficult to compare with modern datasets or the high demands of modern operating rooms. However, 3D reconstruction methods that have been published with results on these datasets have the potential to perform well on newer hardware and are therefore also included in this review. The easy access to suitable datasets in different fields can even make it possible to re-evaluate a developed method as shown in the SERV-CT [17]. Sometimes, methods developed for one application will show promising results when evaluated against another dataset for a different application.

For non-medical stereo applications, the *Middlebury* dataset (MD) and the *Karlsruhe Institute of Technology and Toyota Technological Institute* (KITTI) datasets are the most popular. The Middlebury Dataset is a widely recognized benchmark for evaluating computer vision algorithms, providing datasets for stereo vision, optical flow, and 3D reconstruction. It consists of six different datasets, 2001 [21], 2003 [22], 2005 [23,24], 2006 [23,24], 2014 [16], and 2021 [16]. The KITTI dataset by Menze and Geiger [19] is mainly focused on optical flow applications using their autonomous driving platform AnnieWAY [25]. The latest version is from 2015 and updates the 2012 benchmarks for stereo and optical flow applications while adding a benchmark for scene flow applications [19].

For medical applications, authors can validate their developed algorithms either on their own images and videos or one of the following datasets: the Hamlyn Centre Laparoscopic/Endoscopic Video Dataset, the EndoSlam dataset, EndoAbs dataset, the Stereo Correspondence and Reconstruction of Endoscopic Data (SCARED) sub-challenge of the EndoVis challenge, or the SERV-CT dataset.

The Hamlyn Centre Laparoscopic/Endoscopic Video Dataset provides videos for algorithms working on a binocular and monocular setup [26]. The videos provided are contributed by Mountney et al. [27], Stoyanov et al. [1,28], Lerotic et al. [29], Pratt et al. [30], Giannarou et al. [31], and Ye et al. [32,33]. As of today, in the Hamlyn dataset, only the videos provided by Stoyanov et al. [1] and Pratt et al. [30] come with ground truth data. The ground truth data were generated with the stereo matching software Library for Efficient Large-scale Stereo Matching (Libelas) [34]. Contrary to the MD and the KITTI dataset, the Hamlyn Centre Laparoscopic/Endoscopic Video Dataset does not provide evaluation guidelines or a platform for uploads.

EndoSlam is constructed to design and compare 6-Degrees of Freedom (DoF) pose estimation and dense 3D map reconstruction algorithms. It was published in 2020 by Ozyoruk et al. [35] and the dataset contains recordings and ground truth 3D reconstructions from multiple endoscope cameras of porcine animal organs of multiple cadavers. Image sequences are recorded by a hand-manipulated Panda robotic arm with varying frame rates and changing lighting conditions. In total, the dataset consists of 42,700 frames of which 21,428 are recorded via a High-Resolution Endoscope YPC-HD720P at 1280×720 pixels, 17,978 are recorded via the Low-Resolution 3 in 1 Endoscope Camera at 640×480 pixels, 3055 are recorded via a MiroCam^®^ MC1000-W endoscopic video capsule by IntroMedic (Seoul, Republic of Korea) at 256×256 pixels, and 239 are recorded via a Pillcam^®^ COLON2 double endoscope camera capsule by Medtronic (Minneapolis, MN, USA) at 320×320 pixels. High-precision ground truth data were generated by two commercially available 3D scanners, Artec 3D Eva by Artec Eva (Senningerberg, Luxembourg) and Einscan Pro 2x by Shining 3D (Hangzhou, China). To simulate the problems of transfer learning and domain adaptation the dataset also contains synthetically generated data from a 3D simulation environment. The dataset is not provided with an evaluation toolkit, but the authors suggest determining the RMSE, as they use it for the evaluation of their self-developed algorithm.

In the work of Rau et al. [36] published in 2023, the 2022 EndoVis sub-challenge SimCol3D, the submitted methods, and their results are addressed. The authors argue that a 3D map of the colon could enhance the identification of unscreened colon tissue and serve as a training platform. In their work, the SimCol3D Endovis challenge, three synthetic colon sub-datasets with a resolution of 475×475 pixels are presented together with real video sequences without depth information. The submitted monocular 3D reconstruction methods, their results including RMSE, and published data are discussed in detail. Rau et al. [36] concluded that depth prediction in virtual colonoscopy is robustly solvable, while pose estimation remains an open research question.

The EndoAbs dataset was developed in 2018 by Penza et al. [37]. It focuses on creating a reference point cloud and stereo images of a 3D-printed environment containing abdominal organs. A total of 120 stereo images, with a 640×480 pixels resolution, were recorded by a stereo endoscope consisting of two Ultra Mini CMOS analogical color cameras by MISUMI (New Taipei City, Taiwan). Corresponding reference point clouds were created by a VIVID 910 laser scanner, one of which achieves at a distance of 0.6 m an accuracy of x=±0.22, y=±0.18, z=±0.07 and a precision of 8 µm. The camera-laser calibration error was determined to be 0.43 mm. For determining the error of a 3D reconstruction, the dataset does not suggest an error metric and does not provide a software toolkit. The phantom abdominal organs were printed from 3D models of the liver, spleen, and kidney and also tried to mimic the stiffness and texture of real organs. Varying lighting conditions and smoke are considered in this dataset to create an environment that closely mimics reality. Doctors rated the dataset a respectable average realism rating of 2.7 out of 5 in a follow-up survey.

**Figure 6:**
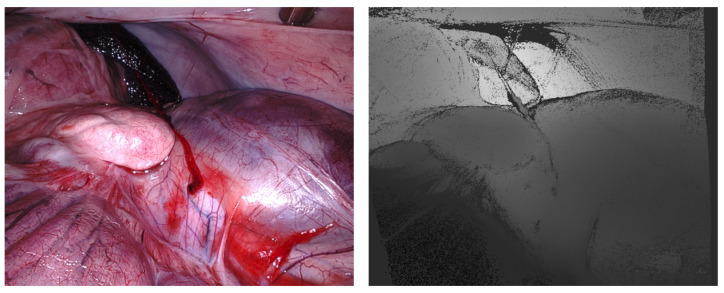
Example image from the SCARED dataset, along with the corresponding depth map [15].

The Stereo Correspondence and Reconstruction of Endoscopic Data (SCARED) EndoVis sub-challenge was presented at the *International Conference on Medical Image Computing and Computer Assisted Intervention* (MICCAI) in 2019 by Allan et al. [15]. It consists of seven stereo-training datasets and two test datasets captured using the da Vinci Xi surgical robot. Each dataset contains four to five unique scene views, called keyframes, of porcine cadavers, as shown in Figure 6. High-precision ground truth data are provided for the first frame of each keyframe using the Structured Light (SL) approach [16]. The SL depth map is rotated and translated based on robot arm/endoscope movements to generate a dynamic sequence with known ground truth values for most of the frames in a keyframe. In addition, stereo calibration parameters were provided in different qualities to simulate improper endoscope calibration or noisy data. The related SCARED publication lists the ranking of all ten participants and gives some details of submitted methods [15].

The Stereo-Endoscopic Reconstruction Validation dataset based on cone-beam CT (SERV-CT) dataset, similarly to the SCARED dataset, aims to provide a stereo-matching dataset for the challenges encountered during MIS. It was developed by Edwards et al. [17] in 2020 to provide a freely available alternative to SCARED. The SERV-CT consists of two sub-datasets, containing eight calibrated image pairs of one of two porcine cadavers together with ground truth data. The images are recorded with a first-generation da Vinci surgical robot by Intuitive Surgical, at a resolution of 720×576 pixels. The CT ground truth data are provided by an O-arm™ Surgical Imaging System by Medtronic. An interventional scanner on the inside of the system generates 3D reconstructions equivalent to CT reconstructed from a rotating X-ray set enclosed within a circular casing. In the second sub-dataset, the authors provide, in addition to the CT data, an RGB surface reconstruction created by the Creaform Go SCAN 20 hand-held structured light scanner. This RGB data allows algorithms to align smooth and featureless surfaces better. To evaluate 3D reconstruction algorithms the SERV-CT provides a toolkit that calculates the RMSE and the Bad3 error. The dataset provides scenes with a range of different tissue types, including smooth surfaces with specular reflection properties, as well as depth variations and occluded surfaces.

A novel 2D-3D registration technique, to register optical mono video sequences with ground truth renderings of a known 3D model, was presented in 2023 by Bobrow et al. [38]. It works by transforming optical images into depth maps with a Generative Adversarial Network (GAN) and aligning edge features with an evolutionary optimizer. The new technique also leverages video information, achieving an average translation error of 0.321 mm and an average rotation error of 0.159° in simulation experiments where error-free ground truth is available. Furthermore, the authors printed a colon with 16 µm resolution on an Objet260 Connex 3 printer by Stratasys (Eden Prairie, MN, USA), molded the print, filmed the resulting mold of the 3D print, and used the proposed 2D-3D registration technique to generate ground truth data. The resulting dataset includes 22 short video sequences registered to generate 10,015 HD video frames of realistic colon phantom models obtained with a clinical colonoscope with ground truth depth, surface normals, optical flow, occlusion, six-degree-of-freedom pose, coverage maps, as well as 3D models. The dataset and registration source code are publicly available.

In 2019, Rau et al. [39] addressed the use of computer-assisted interventions in colonoscopy for early detection and treatment of colorectal cancer. The authors presented a method to generate depth information from monocular endoscopic images by training a conditional GAN dubbed pix2pix. Also, to overcome the lack of labeled training data in endoscopy, they proposed using simulation environments and training the generator and discriminator of the model on unlabeled real video frames to adapt to real colonoscopy environments. The authors reported promising results on synthetic, phantom, and real datasets and claimed that their generative model outperforms discriminative models when predicting depth from colonoscopy images in terms of accuracy and robustness towards changes in domains. With their extended pix2pix model, they reported a mean RMSE of 1.75 mm on synthetic data and a mean RMSE of 16.55 mm ± 0.81 mm on phantom data, which is partially obstructed by markers. In their work, the authors recorded ground truth data from a phantom model via a CT scan and generated synthetic colonoscopy images. Due to the optically overly smooth surfaces of the phantom model, the authors decided to generate another synthetic dataset consisting of about 5000 images using Unity in the same manner as the original training data. The published dataset consists of 16,016 synthetic colonoscopy RGB images with a resolution of 256×256 pixels, varying textures, and corresponding ground truth depth.

### 2.4. Comparability between Contributions

One problem that arises when comparing contributions from different publications and datasets is the usage of various error metrics to determine the accuracy and precision of a method. This problem is exacerbated by many authors who use self-constructed setups for said evaluation and do not publish the data, making replicable testing virtually impossible. In addition to this, there is only a limited number of medical datasets that concentrate on medical endoscopy or laparoscopy. As 3D reconstruction algorithms oftentimes perform well when evaluating against different environments than the ones the algorithm was developed for, we also consider datasets from different fields of research. Nevertheless, it is important to note that each field of research has its own challenges with respect to 3D reconstructions, e.g., deformations and structureless surfaces in the case of medical endoscopy. Additionally, algorithms applied in medical 3D reconstruction need to handle the presence of liquids, such as blood, smoke, and reflections from the endoscope’s light on the tissue. Thus, medical datasets also need to provide such scenes to train or test algorithms on.

The contributions with the best balance between speed and accuracy are compared against each other in Table 2, Table 3, Table 4 and Table 5. The methods have been split into multiple tables, as the contributions are using different datasets for their evaluation and also different lens setups have to be taken into account. For those who use existing datasets, the results can easily be compared against each other as shown in Table 3 and Table 5. All listed contributions in Table 3, have been evaluated on the test images Tsukuba, Venus, Teddy, and Cones of the Middlebury datasets 2001 [21] and 2003 [22]. The listed contributions in Table 5 use the KITTI 2015 dataset and follow a deep learning approach. The medical contributions listed in Table 2 and Table 4 use the RMSE, MAE, and SD to provide the accuracy and precision of their methods. Because some authors use different error metrics and datasets, an objective comparison between their presented methods is not always possible. The same will apply for runtime comparisons when different image sizes are used, different hardware is used, or if there is no mention of the used hardware, to begin with. Nonetheless, while the comparison between the publications is difficult to evaluate, useful information may be extracted. Thus, the information presented in Table 2 and Table 4 may be challenging to compare but is still provided for the sake of completeness.

### 2.5. GPU vs. FPGA

Most of the contributions presented in this overview, use either a Graphics Processing Unit (GPU) or an Field-Programmable Gate Array (FPGA) for computing a disparity map or point cloud. This section will go into detail about the advantages and disadvantages of both platforms.

The GPU architecture allows only limited hardware access [40]. Therefore, it is possible to run older code on new hardware with little to no change to the code itself, for example, when using NVIDIA’s highly parallelizable computing language CUDA [41]. A study in 2012 estimated that for an average post-doctoral employee, a GPU-based implementation of a dense optical flow, stereo matching, or local image features algorithm would take about two months, while developing the same algorithms on an FPGA will take about 12 months for the same person [42]. Real-time stereo-matching algorithms usually do not have a complex structure and, therefore, do not require many branching conditions or complex calculations. Since GPUs outperform FPGAs in tasks with little to no branching conditions and data dependencies [43], it shows why most real-time stereo matching algorithms use GPUs rather than FPGAs. This can be seen in the results of the work by Humenberger et al. [44], and is discussed in Section 5.1.

FPGAs, on the other hand, have a much lower power consumption [45] than GPUs, which are generally not suitable for power-sensitive systems [40]. An FPGA has a power consumption of 5–85 W depending on the application, while a GPU, like the NVIDIA RTX 3090 (Santa Clara, CA, USA), can draw 350–480 W. On a more complex system with many branching conditions, an FPGA is mostly quicker because of its high processing speed; up to billions of operations per second in parallel. The processing speed of an FPGA is faster than other hardware accelerators [40]. The fast processing speeds are achieved because the FPGA can map functions directly to hardware with no external memory access needed since all weights are stored close to the logic elements [46]. Therefore, the GPU will be the best option for real-time stereo-matching algorithms when the best performance is needed, and power consumption is not a priority. If a power-sensitive system is needed, or the algorithm requires many branching conditions, the FPGA will likely outperform the GPU. Due to the fast improvement of GPUs, these advantages will likely further decrease in the upcoming years. In the penultimate paragraph of Section 5.1, this statement is further supported by analyzing data provided by the contributions [44,47,48,49,50].

## 3. Monocular: Passive Methods

This section presents methods that rely exclusively on cameras to perform 3D reconstruction tasks. These methods use the visual input of a camera to acquire landmarks and different perspectives of the observed scene. A 3D reconstruction is performed over time, by identifying invariant robust feature points. Due to the monocular setup, matching algorithms can only be applied to images taken at different times, and therefore monocular systems struggle with strong or rapid camera movements, deforming objects as well as structureless scenes.

The following categorizations are carried out according to the designations provided by the individual authors. This is also the case for Structure from Motion (SfM) and Simultaneous Localization and Mapping (SLAM), which could be merged with regard to the real-time criterion. A summary of passive and active real-time monocular methods evaluated against self-developed datasets can be found in Table 2.

**Table 2:** Comparison of state-of-the-art real-time monocular algorithms evaluated against self-developed datasets. Either the root mean squared error (RMSE) or the mean absolute error (MAE) are determined by the authors to compute the error of the algorithm in millimeters. The methods are listed with the parameters most relevant (see Section 2.2) for real-time monocular methods. The parameters are measurement of accuracy, frame rate, used hardware, and image size. The double line singles out the last publication, as this method, only performs a single-point distance sensing.

Monocular Contribution (Active/Passive, Self Evaluated)	Error in mm	FPS in Hz	Hardware AMD, Intel, NVIDIA (Santa Clara, CA, USA)	Image Size in px
Sfm from tracking [51]	RMSE = 1.9	33	2.5 GHz CPU NVIDIA Quadro FX 570	n.a.
SLAM Dense Surface Reconstruction [52]	RMSE = 2.54	1.6	Intel Xenon 2.8 GHz NVIDIA GTX 970	840×640
ORBSLAM [53]	RMSE = 3–4.1	1.7	Intel i5 3337U 1.8 GHz	n.a.
Endo-Depth- and-Motion [54]	RMSE = 11.02	3.1	AMD Ryzen 9 3900X NVIDIA RTX 2080 Ti	n.a.
VCSEL single point laser distance sensing [55]	MAE = 0.04	5000	n.a	n.a.
3D Scanner Structured Light [56]	MAE = 0.1	30	n.a.	400×400
Infrared coded Structured Light [57]	MAE = 0.12	n.a.	n.a.	640×480
Multispectral Structured Light [58]	MAE = 0.64–0.88	0.02	Intel i7 3770 3.9 GHz	1024×768

### 3.1. Structure from Motion (SfM)

The aim of SfM is to reconstruct an accurate 3D representation, of an object or scene, from a series of 2D images. This is performed by analyzing the projected 2D motion field (motion parallax) of a moving object or scene and tracking corresponding pixels from one image to the next. To aid the reconstruction process, the camera’s motion and 3D position can also be approximated. In monocular setups, due to an unknown baseline between images, the absolute scale of the resulting 3D reconstruction can only be roughly estimated.

SfM is a method that can be used in monocular and binocular setups. With that said, to the best of our knowledge, SfM is currently not represented in binocular stereoscopic endoscopy and therefore the focus of this chapter lies on monocular setups.

SfM is typically performed offline; however, in the work of Sun et al. [51], an online method is presented that relies on both the intrinsic camera parameters and the tracking transformations associated with each acquired image. A magnetic motion tracking device is attached to the endoscope to compensate for the inability to annotate reference points during endoscopic procedures. The device enables an estimation of the position and pose of the endoscope in real time. The reconstruction error is determined by a phantom experiment using a gastric model with 28 markers, which resulted in an RMSE of 1.9 mm. Moreover, by utilizing a GPU, the performance of the proposed method reaches about 30 FPS on a system with a 2.5 GHz CPU, 4 GB RAM, and an NVIDIA Quadro FX 570 (Santa Clara, CA, USA) graphics card.

Recasens et al. [54] approach the SfM problem by using the Monodepth2 network architecture and training procedures [59] in combination with a keyframe-based photometric approach for creating a depth map and tracking the camera pose. With their algorithm, Recasens et al. [54] achieve an average RMSE of 11.02 mm for monocular applications. Using an NVIDIA RTX 2080 Ti (Santa Clara, CA, USA), the algorithm runs with approximately 3.1 FPS.

In the work of Yang et al. [60] geometric structural consistency is taken into consideration and a gradient loss to penalize edge fluctuations is introduced. The proposed method is evaluated across different datasets including the EndoSLAM dataset. Here, the method achieves an RMSE of 66 mm at 120 FPS with a resolution of 320×320 pixels for a sequence of a stomach.

In comparison, an offline SfM approach is utilized in the work of Vélez et al. [61] to reconstruct the surface of a liver. First, the relative pose of the camera in an endoscopic video sequence is reconstructed, using a keyframe-based 5-Point and Perspective-n-Point (PnP) pose estimation. This approach obtains the pose of the camera by minimizing the reprojection error of known 3D points onto the corresponding points. Thereby, it assumes that the camera is calibrated and therefore the intrinsic parameters are known. Then, an initial 3D surface is obtained via optimal triangulation, as proposed by Hartley and Zisserman [62]. With the aid of a Bundle Adjustment (BA) step, where the parameters of the 3D coordinates, relative motion, and the optical characteristics of the camera are simultaneously refined, the initial reconstruction is further optimized. When compared to the ground truth data, which is acquired with a robotic system and reference patterns, a RMSE of 0.19 mm is achieved.

**Figure 7:**
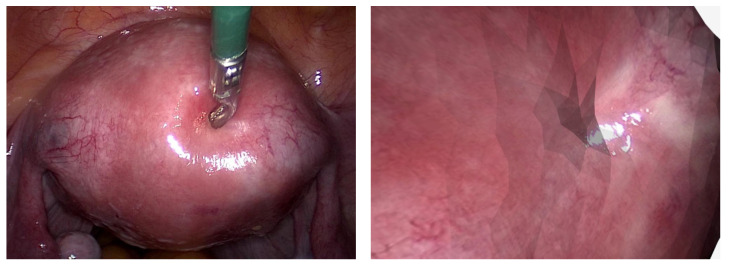
3D reconstruction of an in vivo video sequence from a monocular laparoscope, using the quasi-conformal method as presented by Malti et al. [64].

Similarly, Malti et al. [63] combine both geometric and photometric cues to reconstruct deformable human organs robustly. A visualization of a reconstructed scene is presented in Figure 7. The evaluation of their method features an RMSE less than 0.4 mm on a synthetic deformation model.

The accuracy of the latter approaches shows that when using SfM at an appropriate resolution and working distance, a sub-millimeter resolution can be achieved. Unfortunately, the computational runtime is not documented in [63], so it is not clear whether this method can be used in a daily clinical routine with real-time constraints. To our best knowledge, more recent publications such as the work of Wang et al. [65] in 2017, Turan et al. [66] in 2018, and Widya et al. [67] in 2019, show no further improvements concerning runtime and accuracy. In addition, the authors use a self-constructed dataset which makes an objective comparison challenging.

### 3.2. Simultaneous Localization and Mapping (SLAM)

SLAM has its origin in the field of robotics, where the static world assumption applies, meaning the world to be mapped consists mainly of rigid non-movable, and non-deformable objects. In principle, the method is independent of the sensor technology, however, oftentimes cameras or Light Detection and Ranging (LiDAR) sensors are used. As the name suggests, SLAM refers to the process of estimating the position and orientation of a sensor, while simultaneously creating a map.

Visual SLAM can only determine the translation and rotation of a moving camera up to scale, resulting in point clouds that are missing a conversion factor to determine their absolute scale. Without data fusion with other sensors, missing absolute scale information can further result in a failure to detect scale drift, which in turn can have an impact on loop detections. Nevertheless, efforts have been made to estimate the baseline between consecutive frames to solve this problem, for instance, the work by Rukhovich et al. [68]. They try to estimate the baseline by training a network on different scenes and different baselines.

Here, additional sensors such as Sound navigation and ranging (Sonar) or LiDAR are often used to perform both the localization and the mapping of a robot [69].

With the aid of SfM techniques, this method can also be used in a passive monocular endoscopic setting to create a map of the environment, hence providing a 3D reconstruction. In the work of Mahmoud et al. [53], a real-time 3D reconstruction method, relying solely on an endoscopic image sequence, is presented. The evaluation is performed against Computed Tomography (CT) scans while fixing the endoscope into place relative to the operating table. This is performed while taking into consideration instrument occlusions and tissue deformations. The method uses oriented FAST and rotated BRIEF SLAM (ORBSLAM) [70] to estimate the endoscope position and create a 3D reconstruction of the environment. The oriented FAST and rotated BRIEF (ORB) feature extractor is an alternative to Scale-invariant feature transform (SIFT) and Speeded up robust features (SURF) [71].

Only 24% of the visible point cloud can be mapped, mainly because the ORB feature detector is not able to detect repeatable feature points on soft organ tissue and the BA considers 11–25% of the map’s points as non-rigid. Of these points, 80% are considered in the RMSE calculation, as the remaining 20% are either outliers or points reconstructed outside the field of acquisition of the CT scanner. A brute force approach is used to match acquired point clouds against the ground truth data. After the matching process, a RMSE of 3 mm is achieved, when taking into account only the position of ORB points. When creating a semi-dense map, as presented in the publication, the RMSE drops to 4.1 mm. Although there is no mention of the resolution of the image, an average frame rate of 2.5 FPS, is reported on a not further specified dataset. To achieve this, the authors use an Intel^®^ Core D 3337U CPU (Santa Clara, CA, USA) running at 1.8 GHz and 6 GB of RAM. The authors attempted a dense SLAM based reconstruction in a follow-up publication [72], but with a refresh rate of under 0.17 FPS, the algorithm does not perform in real-time. The hardware is a system with an Intel^®^ i7 running at 3.4 GHz, 8 GB of memory and NVIDIA GTX 680 (Santa Clara, CA, USA).

**Figure 8:**
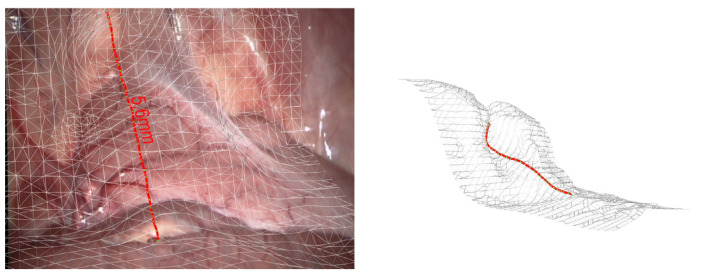
Intra-operative example of a measurement during the 3D reconstruction of an in vivo video sequence. The side-view of the intra-operative measurement example on the right shows that the measurement line closely follows the surface curvature. Reprinted with permission from Chen et al. [52]. Copyright 2024 Elsevier.

A different approach can be found in [52], where a geometry-aware AR framework for depth correct augmentation for intra-operative scenes is presented. The focus of this work is to place virtual objects correctly within the 3D scene in real-time, as can be seen in Figure 8. For this, the camera pose as well as the surfaces of the 3D scene are determined. The developed method is evaluated on the Hamlyn Centre Laparoscopic/Endoscopic Video Dataset [26] and a simulated MIS scene. The evaluation on the Hamlyn Centre Laparoscopic/Endoscopic Video Dataset demonstrates a 3D mesh that shows a good match to the video. This suggests that the method can provide the correct depth information intra-operatively and therefore be advantageous during surgical procedures. However, the authors mention that their method struggles with deformations and instruments, which obstruct large portions of the view. The simulated MIS scene with a realistic human digestive system model is generated with Blender [73]. The RMSE against the ground truth data from the simulation is 1.24 mm for the camera position and 2.54 mm for the surface reconstruction. The 3D surface reconstruction process is implemented without GPU acceleration and reaches 1.7 FPS. The used hardware consists of an Intel^®^ Xeon 2.8 GHz quad-core CPU, 32 GB memory and an NVIDIA GTX 970 graphics card (Santa Clara, CA, USA). A performance increase would likely be possible by upgrading the existing hardware and modifying the algorithm to exploit GPU acceleration.

## 4. Monocular: Active Methods

In this section, we discuss methods, which resort to utilizing active sensors in addition to the camera’s sensors of the endoscope. The sensors are called active as they actively emit a signal, which can then be detected. The needed modifications of the endoscope can lead to an increase in size and/or power consumption. Such modifications are, for example, a Structured Light (SL) projector or a Time of Flight (ToF) sensor. An SL approach works by projecting a known light pattern onto a surface and the measured distortions in the captured projection pattern are used to determine the depth for each pixel. ToF methods typically utilize either pulsed or modulated infrared light and a light detector module that determines the time delay between emitting and detecting and thereby, by taking into account the speed of light, determines the distance to the surface.

For both procedures, the pose of the camera does not need to be known. In the case of the ToF system, a camera is only needed for color information. In addition to that, the usability and stability of the endoscope can be affected as well. A monocular 3D reconstruction is performed using the additional information provided by the extra sensors. A summary of passive and active real-time monocular methods evaluated against self-developed datasets can be found in Table 2.

### 4.1. Structured Light (SL)

**Figure 9:**
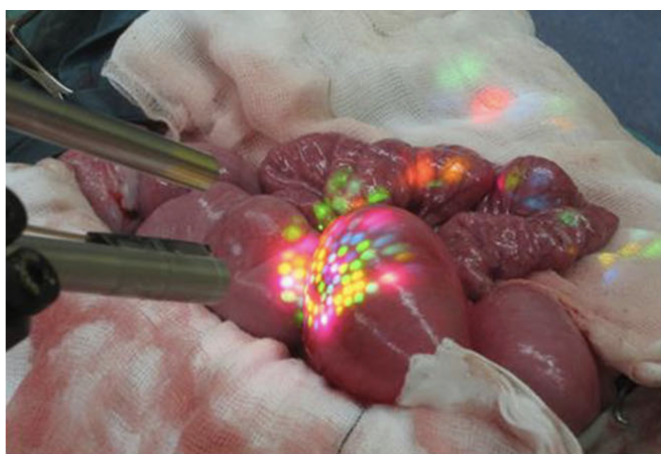
Porcine large bowel under Structured Light (SL). Reprinted with permission from Lin et al. [58]. Copyright 2024 Springer Nature.

The SL approach actively projects a spatially varying or color-coded intensity pattern onto a scene. An example of a color-coded pattern on a surface is depicted in Figure 9. The pattern is generated by a projector or light source modulated by a spatial light modulator. A monocular camera is then used to analyze the projected pattern. If the camera detects a planar surface, the pattern observed by the imaging sensor will be similar to that of the projected SL pattern. In the case of a surface with deformations, the camera records a distorted projection pattern. Based on the distortion, the shape of the surface can be reconstructed [74].

Albitar et al. [75] use a monochromatic light and the spatial neighborhood coding strategy, based on the theory of an M-array with three symbols. The authors modeled a pattern consisting of geometrical primitives. To simplify the search for the relevant neighborhood in the projected pattern, one of the primitives is used to store the local orientation of the pattern. The approach decoded 95% of the detected primitives correctly, even in the presence of spatial occlusions. On an image with a size of 568×760 pixels, the method achieved 12.5 FPS on a P4 2.67 GHz machine.

Another approach is presented by Jia et al. [57]. To acquire the linear dependency between the object depth and pixel shift, they propose a linear fitting algorithm. Using this dependency, the depth information of an object can be derived. The 3D reconstruction of the observed scene is derived by exploiting the Delaunay triangulation. The Delaunay triangulation takes a set of discrete points, where not all points are colinear, and no four points are cocircular, and applies the triangulation in such a way that the circumcenter of the triangle is defined by three points [76]. The authors claim to achieve real-time performance but do not offer data to back up their statement. At a resolution of 640×480 pixels, the method achieves an MAE of 0.12 mm.

**Figure 10:**
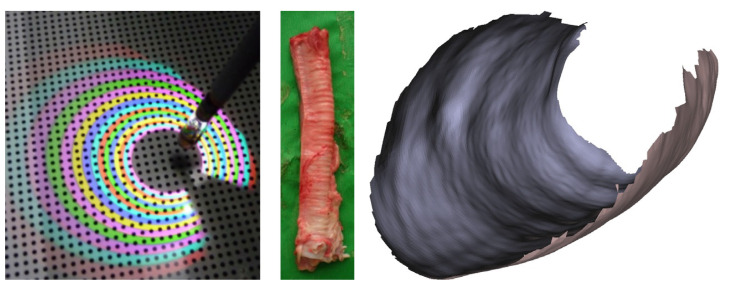
Presentation of the calibration process of the SL method. The depicted lamb trachea was examined in an experiment. The resulting 3D reconstruction is shown on the right. The missing area at the top is caused by the camera connection cable. Reprinted with permission from Schmalz et al. [56]. Copyright 2024 Elsevier.

According to Schmalz et al. [56], a catadioptric camera can be used to detect a circular multispectral pattern in tubular cavities, as seen in Figure 10. The single-shot SL prototype has a diameter of 3.6 mm and a length of 14 mm. The prototype acquires 3D data at 30 FPS and generates approximately 5000 3D points per second. After using the Iterative Closest Point (ICP) algorithm to merge the acquired point clouds, they are smoothed utilizing the method of Vollmer et al. [77] and a surface reconstruction according to Kazhdan et al. [78] is performed. The approach is evaluated at a working distance of about 1.5 cm after calibration with Zhang’s method [13]. When comparing the 3D reconstruction to a CAD model of a known cavity, the MAE between the reconstructed points and the model is 108 µm.

Another multispectral SL approach, using two camera types for capturing the projected pattern, is presented by Lin et al. [58]. The first camera, a DCU 223C by Thorlabs Ltd. (Ely, UK), has a regular color CCD sensor. The other is a multispectral camera, which contains eight different bandpass filters and eight channels in the output image. In terms of calibration, the geometrical camera calibration is applied, as put forward by Zhang [13]. For the evaluation, ground truth is acquired with a NIKON MCAx24+ (Minato City, Tokyo, Japan) handheld 3D scanner, which has a volume length accuracy of ±0.038 mm. Finally, the acquired 3D point cloud is compared to the ground truth using the ICP algorithm [79]. The resulting RMSE, which is derived based on the results presented in [58], equals 1.1 mm.

### 4.2. Time of Flight (ToF)

**Figure 11:**
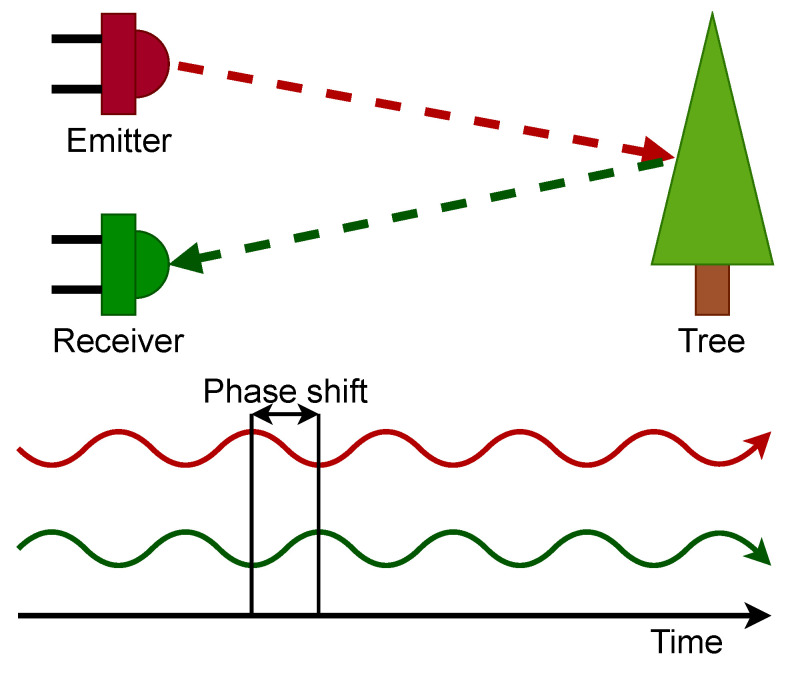
The ToF method uses the phase shift between the emitted and received light pulses to calculate the distance to an object. To determine the distance the phase shift is multiplied by the speed of light and then divided by four times pi, multiplied by the modulation frequency of the emitted light pulse.

The ToF sensors exploit the phase shift between the emitted modulated light pulse and the one received to calculate the distance to an object, as visualized in Figure 11. For short distances, the hardware needs to be able to resolve the gap to the object in picoseconds because of the speed at which light travels. Due to the low cost, low energy consumption, and the possibility to reach 30 FPS [80], ToF sensors are present in multiple applications such as, computer graphics [81], computer-human interaction [82], robotics [83] and autonomous driving [84].

Based on this research, Groch et al. [14,85] develop an algorithm for 3D reconstruction as well. They use a ToF sensor in combination with the SfM approach to improve the accuracy of their algorithm. However, the authors of both papers do not mention the runtime of their algorithms. While using a ToF approach, a MAE of 4 mm ± 1 mm is achieved by both. In their contribution, Groch et al. [14] compare their result to the result of the stereo matching based Hybrid Recursive Matching (HRM) algorithm by Roehl et al. [86], which achieves an MAE of 2.3 mm ± 0.8 mm. The authors conclude that at the time of the publication, ToF methods are inferior to stereo-matching methods with respect to surface reconstruction.

### 4.3. Laser Distance Sensing

**Figure 12:**
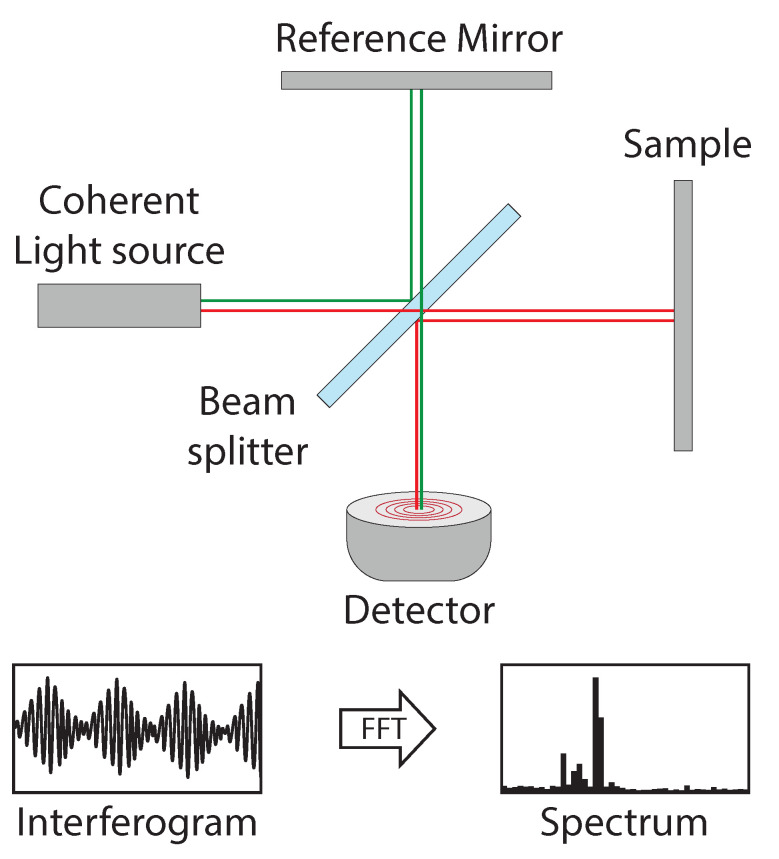
Schematic diagram of a laser distance sensing system based on the Michelson interferometer. The green path depicts the known reference path and the red path length is dependent on the distance to the sample object. The resulting interference on the detector is recorded, and a Fourier transform from the resulting interferogram leads to a spectrum that correlates to the measured depth.

Laser distance sensing was developed based on the Michelson interferometer [87]. The interferometer exploits the difference in optical path length between a known path and an unknown one, as shown in Figure 12. The difference in optical path length is known as the retardation or optical path difference (OPD) and is induced by a beam splitter. An interferogram is obtained by varying the retardation and recording the signal from the detector. To analyze the frequencies and determine the distance to the surface, a Fourier transform is used [88].

Binocular and Monocular methods tend to fail when using triangulation on homogeneous regions due to a depletion of separable landmarks [89]. Lucesoli et al. [90] propose endoscopic single-point laser distance sensing, to measure the distance between the endoscope and the surface. When using a mechanical stepping motor to implement surface scanning, the acquisition time of the method is greater than one second. The authors achieve a resolution of 20 µm at a measurement distance of 20 mm.

To achieve a long measurement range and high scanning speeds, Moon and Choi [91] use a low-cost Vertical Cavity Surface Emitting Laser (VCSEL). This circumvents the mechanical limitations Lucesoli et al. [90] have. The authors cool the laser down by 21 K, to 3 °C, to increase the electric current, which in turn extends the sweep bandwidth by 1.8 nm. This results in a spatial resolution of 135 µm and a range of 100 mm. Building on the idea, Hariyama et al. [92] use four photodiodes and an Semiconductor Optical Amplifier (SOA) to improve both the measuring range and the accuracy. In combination with a galvanometer, the authors are able to create a 3D reconstruction of a scene. A 10 µm accuracy is achieved with the system, which has a measuring range of two meters.

Vilches et al. [55] in contrast, use a Gradient Index (GRIN) lens and a common-path interferometer to reduce the diameter of the system to 500 µm. The authors achieve a precision of 2.7 µm and an accuracy of 40 µm at a measuring distance of up to 50 mm. The surface distance measurement can be performed at a refresh rate of 5000 FPS in an ex vivo setup.

## 5. Binocular: Passive Methods

Passive binocular applications use two camera sensors to create a 3D reconstruction of a scene. Stereo-matching is the most common way to create a 3D reconstruction of a scene with cameras. It works by determining the correspondences between the stereo pairs and using the distance between the matched points to create a disparity map, which is an inverse depth map. The accuracy of the resulting disparity map can be influenced by multiple factors. This includes the camera resolution, the stereo baseline, featureless or reflective surfaces, and harsh lighting conditions [93]. Algorithms using the concept of stereo matching typically follow a global or local optimization approach. Global approaches use the entire image to optimize the result based on a well-behaved energy function. Therefore, they are less sensitive to local outliers when compared with local methods but have higher computational costs [94]. Local functions consider only the local neighborhood of pixels to calculate the matching costs and cost aggregation. The winner-takes-all approach, where the disparity with the lowest cost for each pixel is picked, is typically applied to optimize the matching costs.

Again, the following categorizations are carried out according to the descriptions provided by the individual authors. This is also the case for deterministic Stereo Matching and Deep Learning, while Deep Learning methods can utilize Stereo Matching. A summary of the passive real-time binocular methods evaluated against the MD can be found in Section 5.1, along with a summary of self-evaluated methods. In Section 5.2, a summary of Deep Learning methods evaluated against the KITTI dataset can be found.

### 5.1. Deterministic Stereo Matching

**Figure 13:**
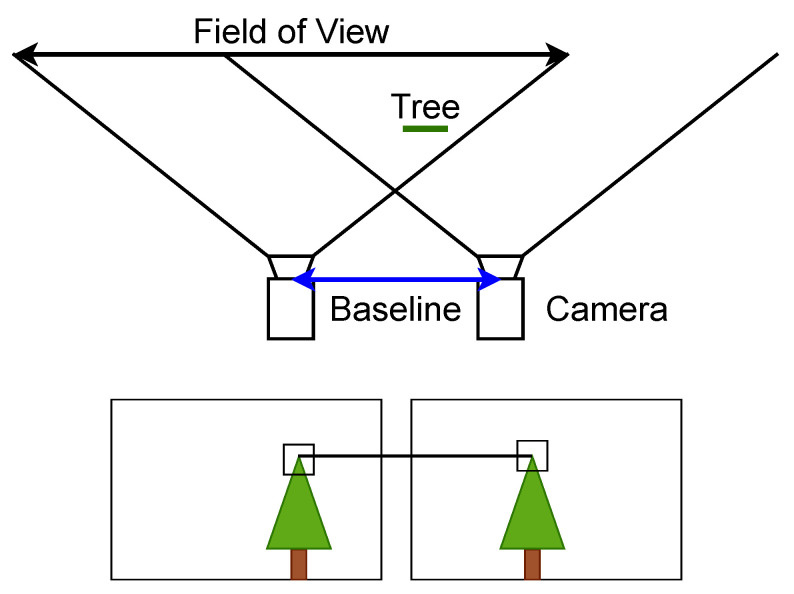
Stereo matching takes advantage of the information provided by two images of the same scene. To determine the 3D position of an object, the corresponding location is identified in both images by either using a correlation-based or a feature-based matching approach. With a known baseline, the resulting disparity can be used to triangulate the 3D position.

Deterministic stereo matching creates a 3D reconstruction from two images of the same scene. To achieve this, two corresponding points along the epipolar line in the images must be found, as visualized in Figure 13. The epipolar line is where the image and epipolar plane intersect, all corresponding matching points can be found on this line. If the images are rectified, the epipolar line runs horizontally to the image plane, reducing the complexity of the search for corresponding pixels. A summary of the passive real-time binocular methods evaluated against the MD can be found in Table 3. Similarly, the self-evaluated passive real-time binocular methods are summarized in Table 4.

**Table 3:** Comparison of state-of-the-art real-time stereo matching algorithms evaluated against the *Middlebury* dataset (MD). The methods are listed with the parameters most relevant (see Section 2.2) for real-time stereo matching. The parameters are Middlebury measurement of accuracy (see Section 2.3, in ascending order), frame rate, used hardware as well as image size and disparity range for the respective stereo pair.

Binocular Contribution (Middlebury Dataset)	Error in %	FPS in Hz	Hardware AMD, Intel, NVIDIA (Santa Clara, CA, USA) Inrevium (Shibuya, Tokyo, Japan)	Image Size in px	Disparity Range in px
Cross-based Support Regions [95]	3.94	49.7	NVIDIA GTX 1070	384×280	16
Guided Image Filtering [96]	5.55	17	NVIDIA GTX 480	640×480	40
Weakly-Textured Scenes [97]	5.78	1	NVIDIA GTX 8800	512×384	48
High-quality Stereo Vision [49]	6.17	31.79	Intel (Altera) EP4SGX230 FPGA	1024×768	96
Two Pass Adaptive Support Weights [98]	6.20	62	NVIDIA GTX 580	320×240	32
Hardware Guided Image Filtering [47]	6.36	60	Inrevium Kintex-7 FPGA	1280×720	64
Line-wise HRM [99]	6.68	13	NVIDIA Tesla C 2070	960×540	n.a.
Real-time BFV [100]	7.65	57	NVIDIA GTX 8800	384×288	16
Belief Propagation [101]	7.69	16	NVIDIA GTX 7900	320×240	16
High-def SM on FPGA [48]	8.20	60	Intel (Altera) EP3SL150 FPGA	1024×768	64
Embedded Real-time Systems [44]	9.73	573.7	NVIDIA GTX 280	320×240	15

**Table 4:** Comparison of state-of-the-art real-time stereo matching algorithms evaluated against self-developed datasets except for the contribution by the Fraunhofer HHI, which evaluated against the SCARED dataset. In the contributions using self-developed datasets, either the root mean squared error (RMSE) or the mean absolute error (MAE) are determined by the authors to compute the error of the algorithm in millimeters. The methods are listed with the parameters most relevant (see Section 2.2) for real-time stereo matching. The parameters are: Measurement of accuracy (in ascending order, RMSE then MAE), frame rate, used hardware as well as image size and disparity range for the respective stereo pair.

Binocular Contribution (Self Evaluated)	Error in mm	FPS in Hz	Hardware AMD, Intel, NVIDIA (Santa Clara, CA, USA)	Image Size in px	Disparity Range in px
Semi-dense Surface reconstruction [102]	RMSE = 3.2	2.64	NVIDIA Quadro K5000	1920×540	n.a.
Semi-dense Surface reconstruction [1]	MAE = 1.06	15	NVIDIA Quadro FX 5800	360×288	n.a.
GPU/CPU Surface reconstruction [103]	MAE = 1.55	30	Intel i7 930 NVIDIA Tesla C 2070	320×240	n.a.
Novel enhancement to HRM [104]	MAE = 2.06	14.5	CPU	n.a.	n.a.
CPU Surface reconstruction [86]	MAE = 2.6	20	CPU	320×240	n.a.
Fraunhofer HHI stereo pipeline [15]	MAE = 3.44 (SCARED Dataset)	45	NVIDIA RTX 3090	1920×1080	n.a.

**Figure 14:**
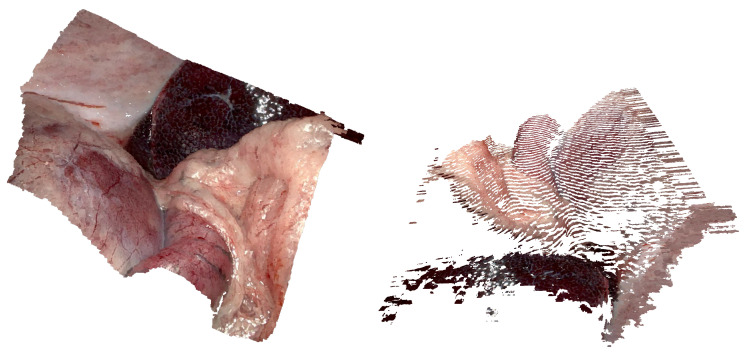
Example of a 3D point cloud from different perspectives generated by a deterministic stereo matching algorithm using images from the SCARED dataset [15] as input.

Given the relative positions of the cameras to one another and the parameters of the camera projection, a triangulation can be performed to determine the 3D position for every corresponding point. An example of a 3D reconstruction using deterministic stereo matching can be seen in Figure 14. Liu et al. [105] show that the minimum working distance should be at least 30 times larger than the baseline between the stereo cameras to generate better stereo images. For stereoscopes with a typical baseline of 4 mm this results in a minimum working distance of about 120 mm which is achievable in a medical environment.

Based on our research, deterministic stereo-matching algorithms follow either a correlation-based or a feature-based matching approach to finding corresponding points. Correlation-based methods use image rectification to optimize the matching process and to compensate for calibration and alignment errors. In doing so, stereo information lies on the same scanline, so that the estimating stereo correspondence search becomes a 1D problem [106]. Feature-based methods use a feature descriptor to find prominent features and a nearest neighbor search to find matching points [107]. Due to faster computation times and denser point clouds, correlation-based methods are more common.

Stereo matching typically concentrates on accuracy, run time, or power consumption. Since we focus on real-time applications in this overview, publications considered here run either in real-time or near real-time.

Newer hardware can allow authors to improve the accuracy of their algorithm, without significantly increasing the original runtime. This aspect is evident when looking at the Cross Based Support Regions (CBSR) methods by Zhang et al. [108]. The contributions [95,100] utilize cross-based support regions for creating a disparity map. A support region’s size and shape are determined adaptively regarding local color values and spatial distances. For a stereo image pair, with a resolution of 384×288 pixels and a maximum disparity of 16 pixels, [100] achieves 57 FPS on an NVIDIA GTX 8800 (Santa Clara, CA, USA). The contribution of Lee and Hong [95] reaches for the same stereo pair 49.7 FPS on a much newer NVIDIA GTX 1070 (Santa Clara, CA, USA). Regarding the accuracy of the approaches, Lee and Hong [95] provide an error in the MD benchmark of 3.94%, while [100] exhibits an error of 7.65%. Thus, the authors use newer hardware to keep a similar frame rate while nearly doubling the algorithm’s accuracy.

Furthermore, it becomes clear that some authors focus on runtime and others on accuracy. This can be seen in the publications [101,109], where the authors improve the idea of Felzenszwalb and Huttenlocher [110] to achieve accurate stereo matching algorithms. The work presented by them in 2004 paves the way for a linear approach to Belief Propagation (BP) in stereo matching. The contribution made it possible to use BP for real-time stereo-matching algorithms. Yang et al. [101] achieve an error of 7.69% on the MD benchmark. Using an NVIDIA GTX 8800 (Santa Clara, CA, USA) and an image with a resolution of 320×240 pixels and a maximum disparity of 16 pixels, they reach 16 FPS. Yang et al. [109] achieve only 0.1 FPS on an image with a resolution of 384×288 pixels and a maximum disparity of 16 pixels. The runtime is not real-time, but the accuracy is significantly improved to an error of 4.19% in the MD benchmark. The authors do not mention what hardware they use in this experiment [109].

Yang et al. [97] also exploit the idea of Felzenszwalb and Huttenlocher [110] with an emphasis on the runtime of the algorithm in their work. The idea of the authors is to use plane fitting in combination with BP. When depth values are believed to be incorrect plane fitting can be used to fit these values to a plane. This is used for example to improve the accuracy in weakly textured scenes [111]. The authors utilize BP to correct potential errors, which are caused by the non-robust process of color segmentation [97]. Using an NVIDIA GTX 8800 and an image of size 512×384 pixels with a maximum disparity of 48 pixels, the authors achieve a frame rate of 1 FPS. In the MD benchmark, the algorithm scores 5.78%, which puts it into the category of the better scoring algorithms in our overview.

While most methods achieve their best performance on a GPU, some algorithms like Hybrid Recursive Matching (HRM) [112] cannot profit as much from the parallelism on the GPU because of their recursive approach. The publications [86,99,103,104], and the algorithm by Allan et al. [15] use the HRM algorithm as a basis. This method exploits the current stereo image pair and the disparity map from the previous image pair to create a disparity map. An initial disparity guess is the width of the image. After that, the disparity estimations are made based on the disparity map from the previous frame. Therefore, the disparity range is not fixed.

To improve the runtime of the HRM algorithm on the CPU [86], it was partially migrated to the GPU in [103]. Due to the recursive nature of the algorithm, parallelization is only partially possible. The runtime is improved by 10 FPS on the same image size when compared to the publication [86]. Roehl et al. [103] also evaluate the accuracy between a GPU and CPU implementation. The CPU implementation achieves an MAE to the ground truth of 1 mm. Due to the changes made to migrate the algorithm to the GPU, the implementation results in a higher MAE of 1.55 mm.

Rosenthal et al. at the Fraunhofer Institute for Telecommunications, Heinrich–Hertz–Institut, HHI, won the 1^st^ place in the category “lowest mean error” and overall 2nd place at the MICCAI 2019 EndoVis sub-challenge “SCARED” [15] organized by Intuitive Surgical. The method is based only partially on the HRM algorithm and is fully migrated to the GPU based on the work by Waizenegger et al. [113]. The stereoscopic 3D reconstruction pipeline consists of two pre- and post-processing steps to improve the overall performance: (1) Apply contrast enhancement using Contrast-limited adaptive histogram equalization (CLAHE) [114] to accentuate tissue structures, which are badly illuminated due to the given co-axial illumination in endoscopic imaging. (2) Usage of a trained depth histogram heuristic, which is based on typical endoscopic working distances, allowing discarding mismatches violating such depth ranges. Besides these assumptions, the disparity range does not need to be set to a constant value. Due to its recursive nature, it adapts automatically to the scene’s depth structure based on the previous estimation step at time t−1 plus a small overhead to compensate for endoscopic camera movement. This means that the start disparity range will be set to the image width for the first initial guess, 1920 pixels in the case of full HD, but it converges quickly to a meaningful disparity range within 15–20 frames. The algorithm achieves an MAE of 3.44 mm on the SCARED dataset. Depending on the scene structure, the method achieves frame rates of up to 45 FPS at a resolution of 1920 × 1080 pixels on an NVIDIA RTX 3090 (Santa Clara, CA, USA).

Kowalczuk et al. [98] and Hosni et al. [96] build on the concept of Adaptive Support Weights (ASW) by Yoon and Kweon [115] in 2005. The ASW method creates a window around each pixel and sums up all pixels inside this window. Before summation, the pixels in the window are weighted based on their color similarity and spatial gradient, compared with the center pixel. To improve the method by Yoon and Kweon [115], Kowalczuk et al. [98] propose using a two-pass approach. This approach results in an error of 6.20%, when compared to the MD. At a resolution of 320×240 pixels and a disparity range of 32 pixels, the authors achieve a frame rate of 62 FPS on an NVIDIA GTX 580 (Santa Clara, CA, USA). The implementation of the ASW algorithm by Hosni et al. [96] accomplishes its accuracy using a guided filter [116], which uses a guiding image to compute the filter output and reduce the errors and outliers created by a cost function. The cost function derives itself by considering the color and spatial gradients. At a resolution of 640×480 pixels and a disparity range of 40 pixels, the authors achieve a frame rate of 17 FPS on an NVIDIA GTX 480 (Santa Clara, CA, USA). An error of 5.55% was achieved when benchmarking against the MD. For better power consumption, [47] implemented their version of the ASW algorithm on an FPGA. The implementation refreshes the output at a frame rate of 60 FPS while processing an image with a resolution of 1280×720 pixels. In the MD the authors achieve an error of 6.36% with their algorithm. The results demonstrate that the optimized hardware approach by Ttofis and Theocharides [47] will suffer in terms of accuracy when compared to the work accomplished by He et al. [116].

As mentioned in Section 2.5, approaches with a focus on power consumption, like the publications [44,47,48,49,50] usually work with FPGAs or microcomputers. A comparison in power consumption between a CPU based-system with an Intel^®^ Core 2 Duo CPU at 2 GHz, a GPU based-system with an NVIDIA GTX 280 (Santa Clara, CA, USA) and an Intel^®^ Q6600 CPU at 2.4 GHz, and a Digital Signal Processor (DSP) based-system with a TI DSK, is given in the contribution of Humenberger et al. [44]. While processing data, the CPU consumes 57 W, the GPU 205 W, and the DSP uses 5 W. When computing the result of the Teddy stereo pair from the MD, the CPU achieves a frame rate of 12.89 FPS, the GPU reaches 105.4 FPS, and the DSP reaches 7.74 FPS. Zhao et al. [50] use the Semi-global matching (SGM) algorithm to compare an NVIDIA Titan X GPU (Santa Clara, CA, USA), with an NVIDIA Jetson TX2 embedded GPU, and with their own optimized implementation for an FPGA. The GPU is the fastest hardware with 238 FPS but consumes 101 W for the computation. The embedded GPU uses only 11.7 W, but only achieves 29 FPS. The authors manage to run the code while consuming 6.6 W on an FPGA and still reaching 161 FPS. Ttofis and Theocharides [47] obtain the on-chip power consumption for the entire system to 2.8 W, while reaching 60 FPS on an 1280×720 pixels and a maximum disparity range of 64 pixels. If power consumption is a concern, the FPGA can reach a high FPS per watt count, and still score an accurate result, as shown in [47,49]. When the highest computational power is needed, such as in an operation scenario, where real-time performance and accuracy are crucial, the GPU will be the best option.

In this state-of-the-art overview, the work of Totz et al. [102] and Stoyanov et al. [1] are some of the few binocular methods that exploit a feature-based matching approach instead of a correlation-based approach. The advantage is that feature-based methods do not require a rectified stereo pair to function. Therefore, this approach turns a 1D search problem along the epipolar line for rectified images into a 2D search problem. While this procedure increases the computational cost significantly, the authors argue that since an existing match is free to move along the image pyramid, which consists of downsamplings of the input image [117], in any direction, the number of successfully matched pixels will be enhanced. The result shown in the contribution by Totz et al. [102] is a sparse point cloud with an RMSE of 3.2 mm when compared to the CT ground truth. On an 1920×540 pixels image, a refresh rate of 2.64 FPS will be possible, when using a NVIDIA Quadro K5000 (Santa Clara, CA, USA). The sparse point cloud shown in the contribution of Totz et al. [102] may not be dense enough for a surgeon to see any details. Totz et al. mention a race condition in their algorithm. Hence, using multiple parallel threads could lead to different results on the same scene when running their algorithm multiple times. An improvement in terms of disparity error and speed, though at a lower resolution of 360×288 pixels, is presented by Stoyanov et al. [1]. The results here show semi-dense 3D reconstructions that are generated at 15 FPS with CUDA on an NVIDIA Quadro® FX 5800 (Santa Clara, CA, USA). For two different 3D reconstructions of a heart, the evaluation results show a disparity error of 0.89 mm ± 1.13 mm and 1.22 mm ± 1.71 mm, resulting in an average of 1.06 mm.

Song et al. [118] argue that despite the numerous proposed Deep Neural Networks (DNNs) approaches, conventional prior-free approaches remain popular due to the lack of open-source annotated datasets and the limitations of task-specific pre-trained DNNs. According to the authors, there was no successful real-time algorithm for non-GPU environments among the prior-free stereo matching algorithms. Therefore, they present the first CPU-level real-time prior-free stereo matching algorithm for MIS. The algorithm makes use of a patch-based fast disparity searching method, together with a coarse-to-fine Bayesian probability, and spatial Gaussian mixed model to evaluate the patch probability at different scales. Experiments demonstrated the method’s capability to handle ambiguities introduced by the textureless surfaces and photometric inconsistencies. When evaluating on an Intel i5-9400 CPU (Santa Clara, CA, USA), on the SCARED dataset, the method achieves an MAE of 2.158 mm with ~14 FPS at a resolution of 1280×720 pixels, and on the SERV-CT dataset, the method achieves an MAE of 3.750 mm with ~15 FPS at a resolution of 720×576 pixels. Please note that the full resolution of the SCARED dataset is 1280 × 1024. The code and a small synthetic dataset of a male colon with 310 stereo pairs at 640×480 pixels are available online.

### 5.2. Deep Learning Photogrammetry

Deep learning algorithms define the state-of-the-art for applications such as robotics, autonomous driving, and speech recognition. In medical stereo matching, given a well-defined dataset they produce similar results, in terms of accuracy, compared to the previously mentioned methods, based on the results of Allan et al. [15]. A summary of the passive real-time binocular methods that utilize Deep Learning and have been evaluated against the KITTI dataset can be found in Table 5.

**Table 5:** Comparison of state-of-the-art real-time stereo matching algorithms evaluated against the *Karlsruhe Institute of Technology and Toyota Technological Institute* (KITTI). The methods are listed with the parameters most relevant (see Section 2.2) for real-time stereo matching. The parameters are KITTI measurement of accuracy (see Section 2.3, in ascending order), frame rate, used hardware as well as image size and disparity range for the respective stereo pair.

Binocular Contribution (KITTI Dataset)	Error in %	FPS in HZ	Hardware	Image Size in px	Disparity Range in px
UASNet [119]	1.64	3.3	2.5 GHz CPU	1242×375	≤150
ACVNet [120]	1.65	5	2 × NVIDIA RTX 3090	1242×375	≤150
LeaStereo [121]	1.65	3.3	NVIDIA V 100	1242×375	≤150
CVCNet [122]	1.74	13.5	2 × NVIDIA RTX 2080 Ti	1242×375	≤150
HITNet [123]	1.98	50	NVIDIA GTX Titan V	1242×375	≤150
HSM [124]	2.14	7	NVIDIA GTX Titan X	1242×375	≤150
DeepPruner [125]	2.15	5.5	4 × NVIDIA GTX Titan X	1242×375	≤150
DispNetC [126]	4.05	15	NVIDIA GTX Titan X	1242×375	≤150
MADNet [127]	4.66	50	NVIDIA GTX 1080 Ti	1242×375	≤150
StereoNet [128]	4.83	60	NVIDIA GTX Titan X	1242×375	≤150
FP-Stereo [50]	7.9	147	Xilinx ZCU102 (FPGA)	1242×374	128
CNN L12 [129]	n.a.	60	NVIDIA GTX Titan X	620×188	n.a.

Deep learning algorithms used in 3D reconstruction can be classified into three categories (1) non-end-to-end, (2) end-to-end, and (3) unsupervised learning algorithms [130].

**Figure 15:**
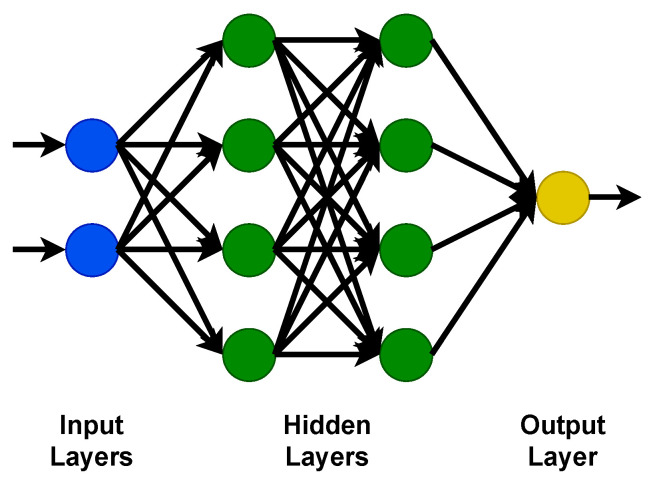
Asimplified structure of a deep learning network such as a CNN. The blue input layers accept the pixels of the stereo images as input. Colored in green are the hidden layers that perform a combination of convolutional operations on the information passed from the blue input layers. To derive the disparity map, the yellow output layer weights the information received by the previous layer.

(1) Non-end-to-end algorithms do not include post-processing to optimize disparity estimation, such as outlier removal, in the network. Therefore, separate post-processing steps are still required to use these algorithms in practical applications [130]. A simplified structure of a Convolutional Neural Network (CNN) network can be seen in Figure 15. According to Zhou et al. [130], non-end-to-end algorithms can achieve a D1-all score of 1 pixel in the KITTI dataset. The disadvantage of this approach is high computational cost, which results in runtimes up to 67 s on an NVIDIA Titan X (Santa Clara, CA, USA) [131]. Also, due to the architecture and setup of the networks, the typical memory footprint is larger than that for deterministic methods. The limited receptive field and the lack of context are further disadvantages. Due to the long runtimes, these methods are not suited for real-time applications.

(2) End-to-end algorithms integrate the whole stereo pipeline into the network. Therefore, the stereo-matching problem becomes more straightforward when compared to non-end-to-end networks.

**Figure 16:**
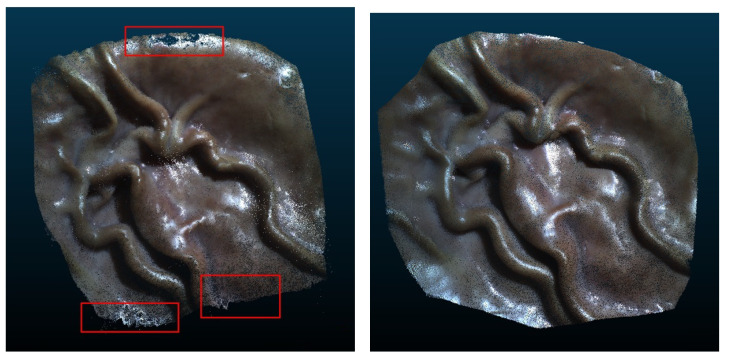
The mosaicked 3D point cloud of a pig stomach obtained by SGBM on the **left**, and by StereoNet presented by Huo et al. [132] on the **right**. Red rectangles indicate areas with outliers in the point cloud that affect the final stitching results due to a rough surface.

Huo et al. [132] present a binocular method that is based on StereoNet [128]. First, an initial disparity map is generated using Semi-Global Block Matching (SGBM). Then, gaps in the disparity map are filled with a self-developed algorithm and a confidence map is generated by comparing the right camera image with a generated right camera image, which is derived from the left camera image and the disparity map. The disparity confidence map is presented to StereoNet as a dataset for training. Thereafter, based on the depth map predicted by StereoNet, the corresponding left image of each depth map is input into the ORBSLAM framework. Color information is obtained from the left camera image, while ORBSLAM is used to estimate the camera pose, and StereoNet is used to acquire depth information. When compared against ground truth data that was generated with the aid of a Einscan-Pro-2x-Plus 3D scanner by Shining 3D (Hangzhou, China) and a phantom as well as a real pig stomach, the proposed method achieves an RMSE of 1.62 mm. The method reaches a framerate of 20 FPS with a resolution of 1280×720 pixels, the resulting stitched point cloud can be seen in Figure 16. It consists of 834,650 points, which ensures the real-time performance of the algorithm.

Tonioni et al. [127] published in 2019 the Modularly Adaptive Network (MADNet) algorithm. With its implementation, the network can adapt to new target domains, such as medical applications, which can be different than the one it was originally trained for. Compared to the state-of-the-art, it is one of the fastest networks available and therefore can run in real-time. When applied on the KITTI dataset it gains a D1-all score of 4.66% with a frame rate of 50 FPS using an NVIDIA GTX 1080 Ti GPU. In the medical domain, the dataset was evaluated on the SERV-CT dataset, on which it scored a Bad3 score of 26.58% ± 18.11% for the first sub-dataset and 38.24% ± 30.26% for the second sub-dataset at 35 FPS using an NVIDIA Tesla V100 GPU. As for the RMSE in each sub dataset, it achieved 4.23 mm ± 1.42 mm for the first one and 6.31 mm ± 3.13 mm for the second one. DeepPruner by Duggal et al. [125] uses a differentiable PatchMatch module. This approach allows the authors to mark a disparity as invalid without requiring a full cost volume evaluation, resulting in quicker runtimes. The minimized disparity range propagated to further refine the disparity range. On the KITTI dataset the algorithm scores a D1-all score of 2.15% with a frame rate of 5.5 FPS using four NVIDIA Titan X GPU (Santa Clara, CA, USA). Edwards et al. [17] evaluated the algorithm on the SERV-CT dataset, on which it scored a Bad3 score of 12.50% ± 7.44% for the first sub-dataset and 19.13% ± 16.95% for the second sub-dataset at 16 FPS using an NVIDIA Tesla V100 GPU. As for the RMSE in each sub-dataset, it achieved 2.91 mm ± 1.71 mm for the first one and 3.21 mm ± 1.31 mm for the second one. Yang et al. [124] use an hierarchical deep stereo matching (HSM) network to develop an approach that can estimate depth quickly. To keep memory consumption low for high-resolution stereo images the authors use an incremental approach to search for correspondences. On the KITTI dataset the algorithm achieves a D1-all score of 2.14% with a frame rate of 7 FPS using an NVIDIA Titan X GPU (Santa Clara, CA, USA). In the publication [17] the HSM network is run on the SERV-CT dataset to evaluate it for medical applications. On this benchmark, it performed as one of the best with a Bad3 score of 8.34% ± 8.31% for the first sub-dataset and 5.46% ± 2.96% for the second sub-dataset at 20 FPS using an NVIDIA Tesla V100 GPU. As for the RMSE in each sub-dataset, it achieved 3.18 mm ± 2.03 mm for the first one and 2.12 mm ± 0.54 mm for the second one. The DispNetC network reaches 15 FPS in the KITTI dataset and a D1-all score of 4.05% [126]. To train the network, an artificially created dataset with more than 35,000 labeled training samples is used. It was also run on the SERV-CT dataset and achieved there a Bad3 score of 40.09% ± 26.41% for the first sub-dataset and 47.87% ± 27.58% for the second sub-dataset at 20 FPS using an NVIDIA Tesla V100 GPU [17]. In the SERV-CT dataset they also calculated the RMSE for each sub-dataset, as 4.58 mm ± 0.76 mm for the first one and 7.07 mm ± 4.70 mm for the second one. As there are no sufficiently large laparoscopic datasets available, ref. [17] used a pre-trained version (against KITTI) of the DispNetC for their evaluation. HITNet runs on an NVIDIA Titan V GPU (Santa Clara, CA, USA) at 50 FPS and manages a D1-all score in the KITTI dataset of 0.5 pixels. As of today, HITNet has the highest accuracy of the above 10 FPS achieving networks concerning the Middelburry-V3 dataset, the KITTI 2012 and 2015 dataset, and the ETH3D benchmark for 3D reconstruction [123]. The UASNet network utilizes an uncertainty distribution guided range prediction model and an uncertainty-based disparity sampler module to achieve a D1-all score of 1.64% at a frame rate of 3.3 FPS on a 2.5 GHz CPU in the benchmark [119].

Cheng et al. [121] developed a method exploiting Neural Architecture Search (NAS) that performs very similarly on the KITTI dataset. The authors achieve a D1-all score of 1.65% at a frame rate of 3.3 FPS on an NVIDIA V 100 (Santa Clara, CA, USA). In the contribution by Xu et al. [120], the authors use Attention Concatenation Volume (ACV) for their network to acquire a D1-all score of 1.65% at a frame rate of 5 FPS on two NVIDIA RTX 3090. Zhu et al. [122] apply super-resolution, which is an upsampling of the input image, to improve the results of their cross-view capture network. Using two NVIDIA RTX 2080 Ti, they attain a D1-all score of 1.74% at a frame rate of 13.5 FPS. StereoNet has a D1-all score of 0.9 pixels in the KITTI dataset and achieves a frame rate of 60 FPS on an NVIDIA Titan X GPU (Santa Clara, CA, USA). The authors also mention a sparse availability of supervised training data. Their aim is to accomplish the same results with an unsupervised learning network [128]. The disadvantages of end-to-end networks are that they require large amounts of data, in the tens of thousands of samples of labeled training data. Unfortunately, there are not many data samples for in vivo procedures with precise ground truth data. Furthermore, the networks are not robust against changes in application because the descriptors must be adjusted for each dataset [130]. In the contribution by Mayer et al. [133], the authors demonstrate that for specific applications, a generalized training dataset is sufficient for an algorithm to perform well later in its application. Therefore, for this approach, a specialized training dataset consisting of real-world data may not be needed.

Chen et al. [134] present a framework for Real-time Scene Reconstruction (FRSR) that is designed to perform a 3D reconstruction of a surgical site in real time. The authors highlight the strong independence of their lightweight encoder–decoder network that performs the disparity estimation. The framework was evaluated via a scene from the da Vinci Research Kit (dVRK) endoscope and more importantly, the SCARED dataset and a self-made clinical dataset captured from an oncology hospital which was also published. The published dataset contains 145,694 image pairs with a resolution of 1920 × 1080 pixels, and calibration parameters are also provided for image rectification and triangulation. The stereo images were recorded during a clinical procedure named Radical Prostatectomy with lymphadenectomy using da Vinci Xi surgical system and a 3D HD video recorder HVO-3300MT by SONY (Basingstoke, UK). The explicit details on how the ground truth in this dataset was acquired are not provided. When evaluating against the SCARED dataset, 25 FPS and an MAE of 2.69 mm ± 1.48 mm and RMSE of 5.47 mm ± 1.34 mm are achieved.

(3) In contrast to end-to-end networks, unsupervised deep learning algorithms approach the disparity estimation problem by minimizing photometric warping errors. The tradeoff is a loss in accuracy [130], which results in an RMSE in the KITTI benchmark of 5.104 mm for well-trained networks while achieving a frame rate of 60 FPS on an NVIDIA Titan X GPU (Santa Clara, CA, USA) [129]. According to Zhou et al. [130], the low quality of unsupervised networks can be traced back to the failure to provide a strong impulse to the network to let it converge correctly when an image reconstruction error occurs.

Self-supervised stacked and Siamese encoder-decoder neural networks to compute accurate disparity maps for 3D laparoscopy are proposed by Bardozzo et al. [135]. These networks are evaluated on three different public datasets, namely the Hamlyn dataset, the SCARED dataset, and the phantom cardiac dataset. Additionally, simulated datasets are generated; however, they are available only upon request and exclusively for research collaborations. The presented networks are capable of producing disparities in real-time on standard GPU-equipped desktop computers. The evaluation results showed that these networks outperformed state-of-the-art methods and achieved an RMSE of 0.29 mm ± 0.05 mm and an MAE of 0.22 mm ± 0.05 mm at ~18 FPS against the SCARED dataset. Finally, a fuzzy post-processing strategy is proposed that slightly improves depth estimation by 0.01 mm at the cost of speed, resulting in ~6.5 FPS.

Large networks need large amounts of domain-specific data for training, which is one of the major drawbacks of deep learning. In recent years research has shown that large amounts of synthetic data can improve the performance of learning-based vision algorithms, which has the advantage that the challenge of generating real-world data for unique scenarios can be avoided. Domain adaptation algorithms are utilized to avoid bigger gaps between simulation and real-world data [35].

Medical devices must be thoroughly validated since human lives may depend on them. Therefore, deep learning and other AI-based systems must pass through a particularly time and resource-consuming validation process. The approval process can be a significant barrier to adopting AI systems in clinical use, according to Benjamens et al. [136]. The FDA proposes a “total product lifecycle” regulatory approach for these methods to remedy that problem. This regulatory approach incorporates continuous learning and improvement of a product in its lifecycle while guaranteeing that safeguards stay in place and the product’s effectiveness is not impacted [137].

## 6. Multi-Ocular: Passive Methods

**Figure 17:**
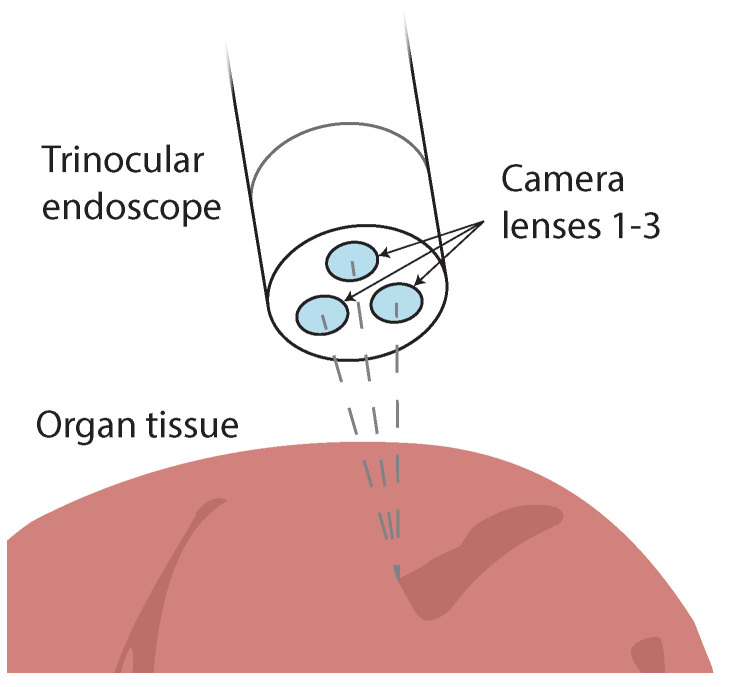
Schematic of a trinocular endoscope observing organ tissue. The dashed lines represent the line of sight for each camera. Stereo matching is performed between all possible camera pairs in order to derive a 3D reconstruction.

Trinocular methods use three camera sensors to create a 3D reconstruction of a scene, as illustrated in Figure 17. Here, the 3D reconstruction process is similar to the one of binocular applications. With three cameras, however, the reconstruction is performed between all possible camera pairs and the depth information is merged, respectively.

Based on the algorithm by Heinrichs et al. [138] a prototype with a triple camera setup is presented by Conen et al. [139]. The results of the trinocular setup are compared to a binocular setup running an SGBM algorithm [140] from the OpenCV library. Conen and Luhmann [89] determine the MAE to the ground truth for the trinocular setup to be 0.29 mm with a SD of 1.14 mm, while the trinocular endoscope is positioned 210 mm away from the object. For the binocular setup, the authors record an MAE to the ground truth of 0.21 mm with a SD of 1.48 mm for the same distance setup. However, in comparison to the binocular setup, which has a typical diameter of 10 mm, the trinocular setup in [139] has a diameter of 14 mm. The authors do not mention the runtime of the algorithm, therefore it has to be assumed that it does not run in real time.

Other multi-ocular approaches use more than three camera sensors or lenses to create 3D reconstructions. Please note that all publications discussed in this section are proof of concepts. Therefore, only limited information is available regarding the subject.

Hassanfiroozi et al. [141] implemented an endoscope using liquid crystal lenses and a single image sensor. The lenses are tuneable by applying a voltage and can switch electronically between 2D and 3D modes. In 2D mode, the lenses pass light rays directly through to an image sensor without focusing, whereas in 3D mode, the light rays are focused on different parts of the image sensor. Even though the stereo basis is small, the lenses can capture an image from different viewpoints such that a 3D reconstruction is possible. The authors focused on the proof of concept and did not further pursue the accuracy or the 3D reconstruction. A year later Hassanfiroozi [142] improved the concept using a multi-liquid crystal three-lens design, which allowed focusing the lenses in 2D mode. The topics of camera calibration, accuracy, and 3D reconstruction were not investigated.

## 7. Conclusions

The growing demand for depth perception in the field of computer vision and technical advancements in recent years have enabled real-time 3D reconstruction methods for medical endoscopy. Some methods require modifications to the existing MIS hardware to function, while others solely rely on the visual input of the camera system. Either way, the fields of navigation, AR, robotics, and many more have greatly benefited from the ongoing research.

Capturing and displaying high-resolution images will always be one step ahead of complex image analysis. As a result, there is currently no abundance of real-time 3D reconstruction methods for high-resolution medical imaging. In addition to this, the comparison between the relevant contributions is challenging. As of today, there is no clear consensus on how to evaluate newly developed methods, and therefore evaluation methods range from open-access datasets to self-constructed ones. In addition to this, authors use different error metrics to evaluate their methods if not specified by a dataset. However, only when publications apply the same dataset and error metric, will an objective and independent comparison between them be possible. Another advantage of using datasets with a specified error metric is the opportunity to re-evaluate a developed method on a dataset from a different field of work. When comparing publications, it becomes clear that some methods being developed for a specific application show promising results when evaluated against another application. One example of this is SLAM, which was initially developed for robot navigation, but also shows promising results in the field of medical endoscopy with minor modifications.

It is good practice to publish the used dataset and hardware together with the runtime, accuracy, and precision of a method to allow for an objective comparison between contributions. If these parameters are not provided, an objective comparison will not be possible.

In our contribution, we aim to provide an objective and comprehensive overview of the current state-of-the-art concerning real-time 3D reconstruction methods for medical applications. The presented methods are first categorized, and subsequently, the most relevant work is compared and grouped into tables, while taking into account different evaluation methods. Furthermore, the evaluation methods used in the compared contributions, including relevant datasets, are outlined. In this, we provide a comprehensive, well-founded starting point, to identify which approach is most feasible for the medical endoscopy application in mind.

## Figures and Tables

**Table 1:** Datasets for 3D reconstruction with ground truth information.

Dataset	Static	Dynamic	Ground Truth (GT) Type	Remarks
EndoSlam	–	*X*	Structured Light	Monoscopic image sequence
SimCol3D	–	*X*	CT data	Monoscopic images & videos
2D-3D Registration	–	*X*	3D Model	Monoscopic videos
Depth from Colon	–	*X*	3D Model	Monoscopic image sequence
Hamlyn	–	*X*	partly available	Mono- & Stereoscopic
Tsukuba	*X*	–	Manual segmentation	The first dataset with GT
Middlebury	*X*	–	Structured Light	Stereo images
Kitti	–	*X*	LiDAR	Stereoscopic videos
SCARED	–	*X*	Structured Light	Stereoscopic videos
SERV-CT	*X*	–	CT data	Stereoscopic images
EndoAbs	*X*	–	Laser	Stereoscopic & Synthetic 3D Models
Phantom Cardiac	–	*X*	CT data	Stereoscopic videos

## Data Availability

The data that supports the findings of this work is available from the corresponding publishers, but restrictions may apply to the availability of this data. Some of the referenced work was accessed under license for the current study, and so is not publicly available.
